# The Molecular Hallmarks of the Serrated Pathway in Colorectal Cancer

**DOI:** 10.3390/cancers11071017

**Published:** 2019-07-20

**Authors:** Fatima Domenica Elisa De Palma, Valeria D’Argenio, Jonathan Pol, Guido Kroemer, Maria Chiara Maiuri, Francesco Salvatore

**Affiliations:** 1 CEINGE-Biotecnologie Avanzate s.c.ar.l., 80145 Naples, Italy; 2 Department of Molecular Medicine and Medical Biotechnologies, University of Naples Federico II, 80131 Naples, Italy; 3 Team of Metabolism, Cancer & Immunity, Centre de Recherche des Cordeliers, UMRS1138, Université Paris Descartes, Sorbonne Université, Université Paris Diderot, 75006 Paris, France; 4 Metabolomics and Cell Biology Platforms, Gustave Roussy Cancer Campus, 94805 Villejuif, France; 5 Pôle de Biologie, Hôpital Européen Georges Pompidou, Assistance Publique—Hôpitaux de Paris, 75015 Paris, France; 6 Department of Women’s and Children’s Health, Karolinska Institute, Karolinska University Hospital, 17176 Stockholm, Sweden

**Keywords:** colorectal cancer, serrated pathway, serrated lesions, serrated polyp, CIMP, DNA methylation, MSI, CIN, serrated adenocarcinoma, gut microbiota

## Abstract

Colorectal cancer (CRC) is a leading cause of cancer death worldwide. It includes different subtypes that differ in their clinical and prognostic features. In the past decade, in addition to the conventional adenoma-carcinoma model, an alternative multistep mechanism of carcinogenesis, namely the “serrated pathway”, has been described. Approximately, 15 to 30% of all CRCs arise from neoplastic serrated polyps, a heterogeneous group of lesions that are histologically classified into three morphologic categories: hyperplastic polyps, sessile serrated adenomas/polyps, and the traditional serrated adenomas/polyps. Serrated polyps are characterized by genetic (*BRAF* or *KRAS* mutations) and epigenetic (CpG island methylator phenotype (CIMP)) alterations that cooperate to initiate and drive malignant transformation from normal colon mucosa to polyps, and then to CRC. The high heterogeneity of the serrated lesions renders their diagnostic and pathological interpretation difficult. Hence, novel genetic and epigenetic biomarkers are required for better classification and management of CRCs. To date, several molecular alterations have been associated with the serrated polyp-CRC sequence. In addition, the gut microbiota is emerging as a contributor to/modulator of the serrated pathway. This review summarizes the state of the art of the genetic, epigenetic and microbiota signatures associated with serrated CRCs, together with their clinical implications.

## 1. Introduction

### The Conventional Model of Colorectal Carcinogenesis and the “Serrated” Pathway

Colorectal cancer is a multifactorial and heterogeneous disease [1]. Most CRCs (75%) are sporadic, whereas about 20% of CRC patients report a family history of the disease. Finally, 3–5% of CRCs are hereditary, with subjects bearing highly penetrant germline mutations that are associated with well-defined cancer-predisposing syndromes such as the hereditary nonpolyposis colorectal cancer (HNPCC), best known as Lynch syndrome (1–3%), or the familial adenomatous polyposis (FAP) (<1%), or again the hamartomatous polyposis syndrome, which displays the lowest incidence (<0.1%) [2].

CRC pathogenesis is due to the progressive accumulation of genetic and epigenetic alterations, some of which being responsible for activating oncogenes or inactivating oncosuppressor genes, that are able to drive the malignant evolution from normal epithelium through early neoplastic lesions (aberrant crypt foci, adenomas, and serrated adenomas) to CRC [3,4]. Such malignant transformation requires up to 15 years, depending on the characteristics of the lesion and on other independent risk factors such as gender, body weight, body mass index, physical inactivity [5].

Neoplastic transformation affecting the colon epithelium is characterized by two distinct morphological pathways of carcinogenesis, namely the conventional and the alternative/serrated neoplasia pathways, each one being defined by specific genetic and epigenetic alterations, typical clinical and histological features and leading to different phenotypes [6,7,8].

**Figure 1:**
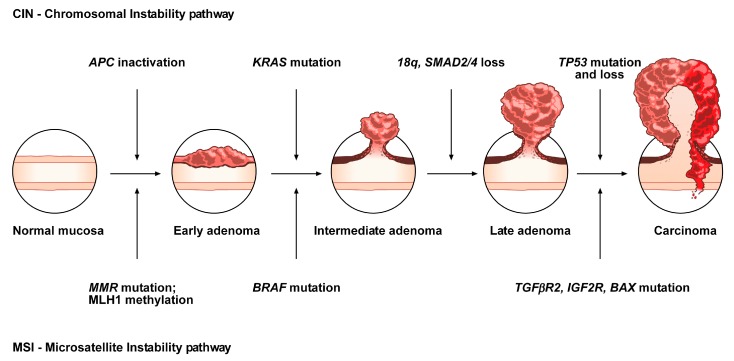
Conventional adenoma-to-carcinoma sequence. The chromosomal instability (CIN) pathway begins with bi-allelic mutations in the tumor suppressor gene *APC* within the normal colonic mucosa. The latter progressively differentiate into adenocarcinoma upon acquisition of additional mutations in the genes *KRAS*, *SMAD4,* and *TP53*, with consequent dysregulation of the Wnt/β-catenin, *MAPK*, *PI3K* and *TGF-β* signaling pathways. Alternatively, the MSI pathway involves an initial alteration of the Wnt signaling that leads to the formation of an early adenoma. Then, *BRAF* mutation followed by alterations of the genes *TGFBR2*, *IGF2R*, and *BAX,* participate in the progression toward the intermediate and late stages of carcinogenesis.

The conventional model, the so-called adenoma-carcinoma sequence, is histologically homogeneous and morphologically characterized the adenoma, including tubular or tubulovillous adenoma, as a precursor lesion [1]. The adenoma-carcinoma sequence is a multistep mutational pathway, in which each histological alteration is the consequence of a molecular dysregulation [9,10,11,12]. At the molecular level, this model recognizes a heterogeneous background, based on two mechanisms of tumorigenesis: (i) chromosomal instability (CIN) or (ii) microsatellite instability (MSI) [13,14,15,16] (Figure 1).

CIN represents the most prevalent form of genomic instability. It is detected in 85% of sporadic CRCs and is frequently observed in distal, rather than proximal, colon cancer sites [15,16]. CIN consists of a gain or loss of all or part of chromosome(s), and is usually associated with mutations in proto-oncogenes or tumor suppressor genes, such as *KRAS* or *APC*, respectively [13,16].

MSI occurs in about 15% of CRCs, predicts a favorable outcome in CRC and can also be detected in the serrated pathway [15,17,18,19]. This genomic instability does not affect chromosomal integrity but consists of an accumulation of insertions/deletions of short nucleotide repeats (microsatellites) that is consecutive to hereditary (5%) or sporadic (10%) alterations in genes involved in DNA mismatch repair (MMR) [14,15].

Although MSI, according to the National Cancer Institute, is frequently determined using a panel of five markers (BAT25, BAT26, D2S123, D5346, and D17S250), a variety of commercially available panels are currently used in most laboratories [20]. Depending on the number of microsatellites associated with these markers, tumors have been subclassified into: (i) high, labeled “MSI”, (ii) low, labeled “MSI-L” or (iii) stable, labeled “MSS” [21]. MSI-L tumors have been regrouped with MSS tumors, due to low differences in their clinicopathological characteristics or in most of their molecular features [22].

Approximately, 3–15% of all CRCs are represented by sporadic forms with MSI [21,23]. Several studies have demonstrated that epigenetic hypermethylation (80% of MSI CRCs), and the consequent silencing and inactivation of the gene *MLH1*, is the event that triggers malignant transformation and determines a high rate of MSI [21,23]. Moreover, mutations in MMR genes (20% of MSI CRCs) can also determine MSI tumors, associated with HNPCC (3% of CRCs) [21,23]. HNPCC is an autosomal dominant disease due to germline mutations in some MMR genes (e.g., *MSH2, MLH1, MSH6, PMS2,* and *PMS1*), causing consequent inactivation of the DNA repair system and the accumulation of mutated microsatellites [24]. In addition, germline deletions in the 3’ end of *EPCAM* result in epigenetic inactivation of the adjacent gene *MSH2* and represent another mutational mechanism responsible for HNPCC (1–3% of HNPCC patients) [25]. HNPCC is not characterized by *MLH1* hypermethylation. Thus, MSI analysis, in addition to *MLH1* evaluation and *BRAF* mutation analysis, is currently one of the first steps for the diagnosis of this disease [24,26].

In contrast to the conventional adenoma-carcinoma pathway, an alternative pathway, featured by the presence of serrated adenomas/polyps as precursor lesions, has been documented over the last 10 years [21,27,28,29,30,31]. It has been estimated that 15 to 30% of all CRCs arise from early neoplastic serrated lesions. These lesions, that are histologically characterized by a “serrated” (or saw-toothed) appearance of the epithelial glandular crypts within the precursor polyps, have long been considered innocuous [31,32,33,34]. Nevertheless, serrated lesions are among the main causes of the “interval” CRCs and are associated with synchronous and metachronous advanced colorectal neoplasia [35,36].

At the molecular level, serrated colorectal lesions rarely present truncating *APC* mutations. The majority of CRCs arising from serrated lesions carry *BRAF* mutations (whose prevalence varies among the different serrated subtypes), while *KRAS* mutations remain less frequent. They are also associated with two pathways, namely MSI and the CpG island methylator phenotype (CIMP), which are involved in genomic instability; the latter being considered as the major mechanism that drives the serrated pathway toward CRC [37,38].

Although the role of *APC* mutations, and the subsequent aberrant activation of the WNT pathway, is fully understood in the conventional adenoma-carcinoma sequence, its role in the serrated pathway remains unclear. To address this issue, the mutational landscape of *APC* in serrated precursors and *BRAF* mutant cancers has been recently explored [39]. In the cited study, even if the WNT pathway was notably activated in dysplastic serrated lesions and *BRAF* mutant cancers, it was not due to truncating *APC* mutations, suggesting the existence of alternative mechanisms of activation of the WNT signaling. Moreover, the role of missense *APC* mutations, which are relatively frequent in serrated lesions and *BRAF* mutant cancers with MSI, should be further investigated in the serrated pathway.

Overall, CRCs have been classified into five molecular subtypes based on their MSI and CIMP status, among which the three following signatures describe serrated lesions [21]:

- CIMP-H, *MLH1* methylated, MSI, *BRAF* mutated lesions, known as sporadic MSI;
- CIMP-H, *MLH1* partially methylated, MSS, *BRAF* mutated lesions;
- CIMP-L, *MGMT* methylated, MSS, *KRAS* mutated lesions.

However, alternative molecular classifications have been proposed based on recent findings, such as those defined by the CRC Subtyping Consortium (CRCSC) or by Fennell et al. [40,41,42].

The heterogeneity of serrated lesions and the presence of morphological features shared with different subtypes, make difficult the accurate CRC classification during the diagnostic process and also the physiopathological interpretation of the observed lesions. In addition, serrated lesions with a distinctive endoscopic appearance are more difficult to detect compared to conventional lesions. In fact, detection of serrated lesions, particularly those located in the proximal colon, is difficult and endoscopist-dependent [43,44]. Therefore, characterization of molecular markers specific for each CRC subtype may improve the identification of CRCs arising from this alternative pathway, and consequently support the diagnostic process as well as the clinical decision-making.

This review will focus on the genetic and epigenetic features involved in the development of the serrated lesion patterns and their relationship with CRC development. In particular, since the methylation status has emerged as a relevant biomarker for CRC classification/prognostic evaluation and is also involved in the progression of serrated lesions toward carcinoma, we will first describe the methylation alterations related to CRCs. Then, the genetic and epigenetic alterations related to serrated precursor lesions and to the serrated pathway will be detailed, with particular attention on one of the endpoints of the serrated pathway, namely the serrated adenocarcinoma.

We will also report the recently discovered superficially serrated adenomas subtype as well as the association between gut microbiota and the serrated pathway. Finally, the clinical implications of the serrated colorectal pathway will be discussed.

## 2. Histopathological and Endoscopic Features of Serrated Colorectal Lesions

**Figure 2:**
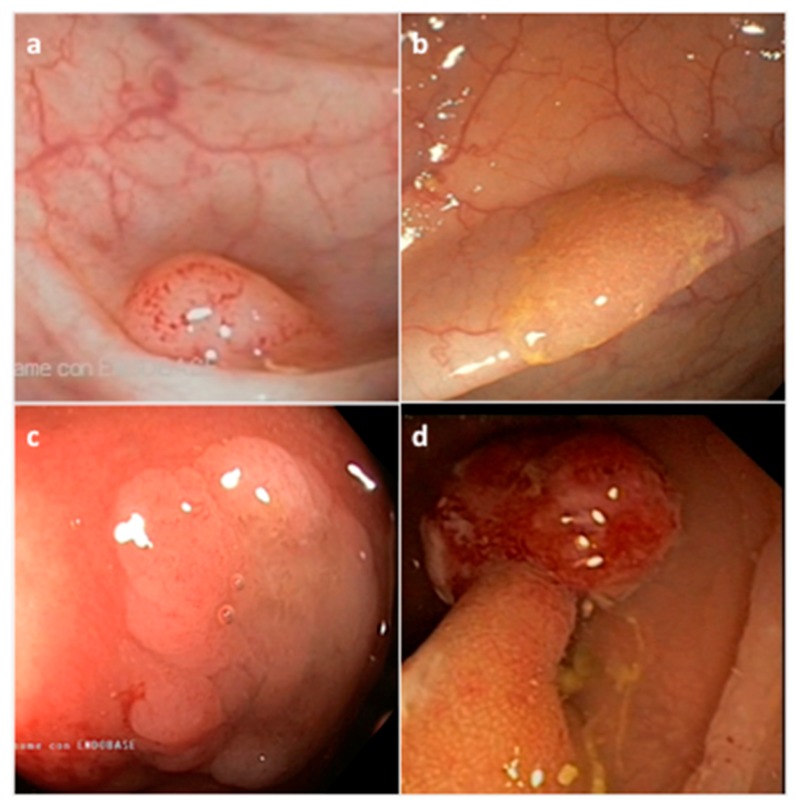
Representative endoscopic appearance of serrated lesions of the colorectum. (**a**) Hyperplastic polyp; (**b**) Sessile serrated adenoma/polyp; (**c**) Sessile serrated adenoma/polyp with dysplasia; (**d**) Traditional serrated adenoma. (Courtesy of Prof. Dr. Giovanni D. De Palma, University of Naples Federico II, Naples, Italy).

Serrated neoplasia of the colorectum represents one of the CRC subtypes [45]. They are histologically classified by the World Health Organization (WHO) into three morphological categories: (i) hyperplastic polyp (HP), (ii) sessile serrated adenoma/polyp (SSA/P) with or without cytological dysplasia (SSAD), and (iii) the traditional serrated adenoma/polyp (TSA) (Figure 2) (Table 1) [46]. The serrated subtypes, identified by their cytological characteristics and lesion area, have a distinct endoscopic appearance, share some histological features, and are unique at the biological and molecular levels [34,47].

HP precursor lesions are the most frequent polyps (80–90%) and mostly remain benign (Figure 2a) (Table 1) [48]. HPs may be flat or sessile, are preferentially located in the distal colon, show a smaller size (< 5mm) than other subtypes, and remain hardly detectable by endoscopic exam. Based on the epithelial mucin content, HP lesions can be histologically subclassified into microvesicular HP (MVHP), goblet cell HP (GCHP), and mucin poor HP (MPHP) polyps (Table 1) [28,30,34]. Although the frequency of each HP subtypes is variable, MVHPs are the most common, while MPHPs are the rarest form of HPs [49,50].

SSA/Ps, which account for 15 to 20% of all serrated polyps, especially develop in the proximal colon, are pale lesions that can be either sessile or flat, with a variable size usually larger than 5 mm in diameter (Figure 2b) (Table 1) [48,51,52]. SSA/Ps can develop either as primary tumors or evolve from hyperplastic polyps. SSA/Ps lesions cannot be easily distinguished from MVHPs, however, MVHPs larger than 10 mm in diameter can be considered clinically equivalent to SSA/Ps. Additionally, SSA/Ps can be subclassified according to the absence or presence of dysplasia (SSAD); the latter being detected in about 0.20% of all serrated lesions (Figure 2c) [51]. Overall, by combining both serrated and dysplastic features, SSADs consist of advanced lesions that usually evolve rapidly toward carcinoma.

TSA lesions are the rarest form of colorectal serrated polyps (1–6%) (Figure 2d) (Table 1) [34,48]. TSAs, that arise either from HPs or SSA/Ps, are precancerous sessile or, more often, pedunculated polypoid lesions, which preferentially develop in the distal colon and rectum, and show a larger size (>5 mm) than HPs [50]. A less aggressive variant form of TSA is the filiform serrated adenoma [53]. TSA can also be subclassified according to the presence of dysplasia that can be of two types: the well-known “adenomatous dysplasia” and the less frequently observed “serrated dysplasia”, which is related to the serrated pathway. The latter can be graded as low- or high-grade dysplasia depending on the absence or presence of cytological and architectural atypia, respectively. Recently, it has also been evidenced that TSA can co-exist with other lesions such as HPs, SSA/Ps and tubulovillous adenomas [54]. Usually, only TSA with serrated dysplasia develops into invasive carcinomas.

## 3. CpG island Methylator Phenotype, an Epigenetic Signature of Serrated Colorectal Lesions

Methylation is one of the epigenetic mechanisms that regulate gene expression [55,56]. Precisely, it consists of biochemical modification of the DNA that is catalyzed by DNA methyltransferases and results in the covalent addition of a methyl group (CH_3_) to the carbon 5 of the cytosine ring of CpG islands (Cytosine-Guanine dinucleotide group) located in gene promoter regions [56].

DNA methylation (mDNA) is a regulatory mechanism used by cells to silence the expression of a target gene, such as a tumor suppressor gene. In particular, when a CpG site is methylated within the promoter region of a gene, its transcription is inhibited. Aberrant DNA hyper/down-methylation is involved in several diseases, including cancer [57]. Interestingly, assessing the methylation status of specific genes could be useful in clinical practice. Indeed, it could represent a biomarker for the detection or monitoring of specific diseases such as CRC, and more particularly of the serrated lesions that are associated with a CIMP signature [58,59,60]. For instance, a recent study reported on a positive correlation between CIMP and older age in SSA/Ps, indicating a lower risk of developing a malignancy in young patients [61].

It has been estimated that 20–30% of all CRCs harbor the CIMP phenotype [62,63,64]. In serrated lesions, although the CpG island methylation can occur in crypt foci and small HPs, the normal mucosa of patients affected by hyperplastic polyposis (HPP) or CRC has demonstrated promoter hypermethylation in some specific genes, accompanied with genetic predisposition to such epigenetic event [22,65,66].

CIMP represents a distinct phenotype in CRC, with specific clinical, pathological and molecular features [64,67]. The origin of CIMP, including its association with *BRAF* mutation remains controversial. Several hypotheses, such as the implication of *BRAF* or *MAFG*, have been explored [68,69]. Using a mouse model of colon derived-organoids, Tao et al. have recently demonstrated that aging-like spontaneous methylation of multiple genes produces a cell that avoid *BRAF*-induced senescence, and predisposes colon cells to oncogene-driven carcinogenesis [70].

CIMP can be graded as low (CIMP-L), high (CIMP-H) or negative (CIMP-0), depending on the degree of simultaneous hypermethylations occurring in several CpG islands located near the promoter region of tumor suppressor genes (Table 2) [71,72].

CIMP-H, which is preferentially localized in the proximal colon, occurs more frequently in females and in older age, and shows the poorest prognosis compared to CIMP-L [62,73]. At the molecular level, CIMP-H is often a MSI tumor and is featured by the inactivation of WNT/β-catenin pathway, high *BRAF* and low *TP53* mutation rates. In comparison, CIMP-L is associated with *KRAS* mutations while CIMP-0 is characterized by frequent mutations in *TP53* (Table 2) [62,74].

Although the categorization and the cut-off between the CIMP high and low phenotypes have been modified several times over the last years, there are currently two panels of genes used to define the CIMP phenotype. The first one was described by Toyota and considers *p16*, *hMLH1*, *MINT1*, *MINT2*, and *MINT31*, while the second panel has been described by Weisenberger and takes into consideration *CACNA1G*, *IGF2*, *NEUROG1*, *RUNX3*, and *SOCS1*. These panels allow to verify the transcriptional inactivation of the genes mentioned and involved in the carcinogenetic development [75,76].

As described above, the hypermethylation status of the genes listed in the CIMP panels can be used as a biomarker for prognosis, prediction, diagnosis and response to chemotherapy of CRC. For instance, evaluation of the methylation status of the *MLH1* promoter is nowadays added to genetic testing for the hereditary Lynch syndrome, usually *MLH1* negative [24].

Another epigenetic marker is O(6)-methylguanine-DNA methyltransferase (*MGMT*) [21,77]. Hypermethylation of the *MGMT* promoter is preferentially associated with *KRAS* mutations, CIMP-L, MSS phenotype due to the overloading of the DNA mismatch repair system. The methylation and the loss of *MGMT* are highly variable among the CRC subtypes, occurring usually in 22% of HPs, 25% SSA/Ps, 16–22% serrated adenoma, and 50% of serrated adenocarcinoma, but is less frequently associated with HNPCC (6%) [77].

Different methylome-based studies, as the recent one of Parker et al., have identified candidate markers for DNA methylation in serrated CRC, notably for the pre-colonoscopy detection of precancerous lesions [78]. These biomarkers of precancerous serrated lesions include *BMP3*, *NDRG4*, *ANXA10* and are analyzed through stool DNA tests. They allow distinguishing between SSA/Ps and conventional adenomas [79,80].

Genome-scale mDNA studies have also been applied to better characterize the DNA methylation subtypes in CRC. In light of the identification of four DNA methylation–based subgroups of CRC, five clinically and molecularly distinct CIMP subtypes in CRC (CIMP-H1, CIMP-H2, CIMP-L1, CIMP-L2 and CIMP-neg), based on a comprehensive methylome-based study and validated in a larger cohort of CRC data from TCGA, have also been identified recently [42,81]. In this new scenario, the major difference between this latter and the current CIMP classification is the dichotomization between CIMP-H1 and -H2 cancers. While CIMP-H1s are characterized by female gender preponderance, and proximal colon location for *BRAF* mutated tumors, CIMP-H2s are preferentially located in distal colon and frequently harbor *KRAS* mutations.

At present, there are several gene methylation analysis methods to define the CIMP-CRC profile, such as the non-quantitative methylation-specific PCR (MSP) technique or the high-throughput quantitative methylation PCR MethyLight assay. In addition, pyrosequencing, InfiniumMethylation bead array, MassARRAY, methylation-sensitive high-resolution melting (MS-HRM), next generation sequencing (Methyl-Seq) and combined bisulfite restriction analysis (COBRA) methods can also be used.

Although the comprehension of the epigenome landscape needs to be extended, the identification of specific epigenetic alterations driving tumor progression has become crucial to better classify the heterogeneous molecular subtypes of CRC. Ultimately, it will facilitate the development of innovative epigenome-based personalized medicine strategies.

## 4. Molecular Features of the Serrated Colorectal Precursor Lesions

### 4.1. Hyperplastic Polyps

As described above, hyperplastic polyps can be histologically subclassified into MPHP, GCHP and MVHP lesions (Table 1). The endoscopic diagnosis between these subtypes, and furthermore between HPs and SSA/Ps, is difficult and may be supported by the detection of specific biomarkers.

MPHP is the rarest form of HP and is not well described; in fact, to date, it has only been associated with CIMP-H (Table 3). MVHP and GCHP are the most common HP subtypes. At the molecular level, MVHP is particularly characterized by *BRAF V600E* mutation and CIMP-H (Table 3); for that reason, it is considered a precursor of SSA/Ps [17,82].

GCHP is linked to *KRAS* mutations (often missense substitutions at glycine codons 12 or 13) and CIMP-L (Table 3) [19,83]. Mutations in *BRAF* or *KRAS*, that rarely coexist in CRC, constitutively activate the MAPK signaling pathway, which is involved in the regulation of several cellular processes, and inhibit the apoptosis mechanism, thus supporting tumor cell proliferation.

To shed light on other molecular features of HP lesions, early markers of potentially malignant serrated precursor lesions have been identified, such as *MUC5AC* [84,85]. MVHP and SSA/P lesions present an hypomethylated *MUC5AC* when compared to GCHPs. Interestingly, this gene hypomethylation occurs early in the serrated pathway, gradually increasing from MVHP to SSA and SSAD, and is particularly related to lesions with *BRAF* mutations, CIMP-H and MSI. Thus, the epigenetic alteration of *MUC5AC* could be a potential marker to evaluate the malignant evolution of serrated precursor polyps.

### 4.2. Sessile Serrated Adenoma/Polyps

At the molecular level, SSA/Ps are mainly characterized by *BRAF* mutations, MSS, CIMP-H and unmethylated *MLH1* (Table 3) [17,86]. Other molecular characteristics of sessile serrated lesions, as well as a subtype-specific gene signature, have been explored and, in some cases, identified based on epigenetic and transcriptomic approaches. An example is the recent molecular characterization of SSA/Ps in a large African American cohort, in which the over-expression of *FSCN1* and *TRNP1* seemed to segregate with race [87].

The formation of SSA/Ps has been associated with the tumor suppressor gene *SLIT2*, who is down-expressed in SSA/Ps compared to TSAs/adenomas/normal tissues as a result of promoter hypermethylation and loss of heterozygosity [88]. The high rate of *SFRP4* methylation in SSA/P compared to the corresponding adenoma series has also been evidenced [86]. *CTSE*, *TFF1* and *ANXA10* were identified as potential clinical markers of SSA/P lesions [89,90,91]. In particular, ANXA10 expression levels significantly increased at the gene and protein levels in SSA/Ps in comparison to MVHPs [90,92]. *Hes-1*, a downstream target of the Notch signaling pathway which involved in intestinal development, has also been described as a SSA/P-specific biomarker due to its immunohistochemical (IHC) loss of expression in SSA/Ps compared to HPs or normal colonic mucosa [93].

In a large-scale study, differentially expressed genes and immunohistochemical markers were also identified when comparing SSA/Ps to controls [94]. In particular, among the 1294 genes identified, *VSIG1* and *MUC17*, were uniquely and significantly increased in SSA/Ps with respect to controls/HPs/adenomas, thus evidencing a molecular signature specific of the polyps and the involvement of different molecular pathways across distinct CRC lesions.

Recently, a platform-independent approach was adopted in order to differentiate SSA/P from HP lesions [95]. SSA/Ps have been characterized by a specific molecular profile of up-/down-regulated genes involved in the inflammatory process, immune response, epithelial–mesenchymal transition (EMT), extracellular matrix (ECM) interaction, cell migration and cell growth. This profile defines the malignant potential of SSA/P lesions and allow to distinguish them from HPs.

As for MVHP polyps, SSA/P immunohistochemically expresses MUC2, MUC5AC and MUC6 [84,96]. The role of MUC6 was evaluated in SSA/Ps lesions and is controversial [84]. Nevertheless, MUC6 was assessed to be a supportive immunohistochemical marker to differentiate SSA/Ps from TSAs [97,98].

### 4.3. Sessile Serrated Adenoma/Polyps with Dysplasia

The main molecular characteristics found in SSADs are *BRAF* mutations, a nuclear β-catenin accumulation, CIMP-H and MSI as a consequence of *MLH1* gene silencing due to promoter hypermethylation (Table 3) [99]. Although the loss of *MLH1* expression is related to the development of dysplasia, *MLH1* inactivation can also be detected in SSA/P non-dysplastic crypts, indicating the putative biomarker role of *MLH1* in predicting dysplastic progression of these polyps [100].

Moreover, it has been demonstrated that SSAD lesions can be also associated with MSI, harboring different genetic alterations [99]. In particular, MSI lesions are characterized by a high mutational rate of *FBXW7* and the loss of *MLH1* expression, while MSSs display *TP53* mutations without *FBXW7* mutations. Thus, *FBXW7* alterations can be correlated to the progression of MSI serrated lesions in CRC.

SSAD polyps, and particularly the progression of SSA/P lesions toward dysplasia, can also be characterized by alterations in WNT signaling-associated genes like protein-truncating mutations of *RNF43*, *APC*, *ZNRF3* and the hypermethylation of *AXIN2* and *MCC* [99,101,102,103]. Furthermore, frameshift mutations in *RNF43* are frequently present in patients with *MLH1*-deficient SSA/P with dysplasia [101].

Genetic variants of DNA-regulatory elements, such as single nucleotide polymorphisms (SNPs), can influence gene expression and be associated with cancer risk. An example is the well-known CRC-associated rs1800734, or MLH1-93G>A, which can be detected in the promoter region of *MLH1*. This SNP enhances *DCLK3* expression which in turn promotes CRC development [104]. MLH1–93 G/a polymorphism has been significantly associated with *MLH1* hypermethylation in SSAD lesions compared to TSAs, and represents a putative risk factor for *MLH1* promoter methylation [105].

### 4.4. Traditional Serrated Adenoma/Polyps with and without Dysplasia

TSA is an enigmatic subtype due to its heterogeneous molecular features [54,106]. TSAs can harbor *KRAS* or *BRAF* mutations, or neither, and can be CIMP-low or high, and MSS (Table 3) [107]. In contrast with SSA/Ps, TSA lesions do not show *MLH1* promoter hypermethylation but can present *MGMT* hypermethylation (Table 3) [108]. Evaluation of the mutational status of WNT genes in TSA lesions has identified precursor- and TSA-specific alterations that elucidate the mechanism involved in the genetic transition from precursor polyps to TSAs [109]. PTPRK-RSPO3 fusion and mutations in *PTEN*, *RNF43*, *APC*, or *CTNNB1* are genetic features of TSAs [110,111].

Several epigenetic biomarkers have also been associated with TSA. An example is *SMOC1* which is down-expressed, due to methylations in its promoter region, in TSAs as compared to SSA/Ps [112]. In particular, *SMOC1* methylation increases during TSA development and has been correlated with *KRAS* mutation and CIMP-L. In addition, low expression of the SMOC1 protein in TSA can be verified by IHC assay, supporting its exploitation as a TSA-specific diagnostic biomarker.

The transition of TSAs toward dysplasia is characterized by nuclear β-catenin accumulation, *TP53* mutation and *p16* inactivation, as detailed below [21]. TSA-HGDs are usually characterized by CIMP-H, *BRAF* mutations and MSS (Table 3). The high rate of differentially methylated region in the promoter P0 of *IGF2* gene (IGF2 DMR0) and hypomethylation of *LINE-1* are two epigenetic biomarkers of TSA-HGD [113]. *LINE-1* hypomethylation in CRC has been associated with early age onset and family history of CRC. In several studies, *LINE-1* hypomethylation has been inversely correlated with MSI and CIMP-H phenotypes. Interestingly, the methylation level of *LINE-1* has recently been assessed from plasma-circulating cell-free DNA, thus comforting a putative role as a preventive biomarker in CRC [114,115].

## 5. Serrated Adenoma to Carcinoma Sequence: Initiation and Progression

**Figure 3:**
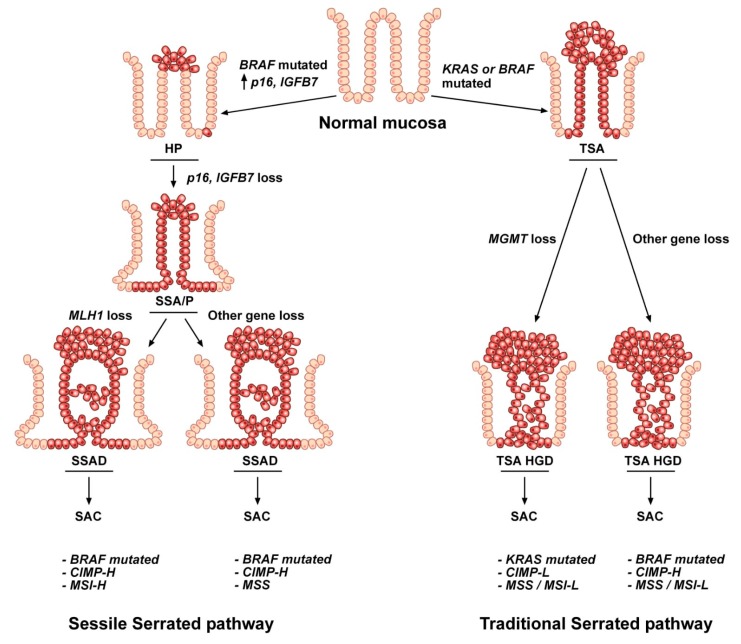
Representation of the two serrated pathways. The sequence leading to serrated Colorectal cancer (CRC) can occur in two different molecular pathways, the sessile and the traditional serrated routes. The tumorigenic process involves progressive accumulation of specific genetic and epigenetic hits affecting normal colon epithelial cells. In the sessile serrated pathway, the transformation of the normal mucosa begins with *BRAF* mutations, followed by *p16* and *IGFBP7* promoter hypermethylation and the consequent progression toward serrated adenocarcinoma, mainly through *MLH1* epigenetic alterations. In contrast, the traditional serrated pathway involves *KRAS* or *BRAF* mutations in normal colon cells which, together with *MGMT* or other gene methylation alterations, malignantly evolve toward traditional serrated adenoma (TSA) high-grade dysplasia and serrated adenocarcinoma (SAC).

The colorectal carcinogenetic mechanisms that drive malignant transformation from the normal colon mucosa to serrated polyp, then to carcinoma can be distinguished in traditional and sessile serrated pathways based on the lesions involved in the oncogenic process, TSA and SSA/P respectively, and on different molecular features: *BRAF* or *KRAS* mutations, hypermethylation of CpG islands and MSI status (Figure 3) (Table 3) [29,33].

Cancers arising through the traditional serrated pathway are predominantly localized in the distal colon and rectum. This pathway is the most controversial due to the molecular heterogeneity of the TSA lesions. The carcinogenetic mechanism that drives malignant transformation is *KRAS* or *BRAF* mutation in normal colon which consequently evolves toward a TSA lesion [29]. This pathway is characterized by a CIMP phenotype and is rarely associated with *MLH1* hypermethylation (Table 3).

TSAs can progress toward dysplasia via two different molecular pathways [116]. Usually, TSA lesions which harbor *KRAS* mutations evolve toward conventional adenomatous dysplasia, while TSAs, which display *BRAF* mutations progress toward serrated dysplasia. Moreover, both dysplastic lesions can advance toward HGD due to aberrant promoter methylation of some genes. Among these latter, *MGMT* is usually associated with CIMP-L and MSS status (Figure 3). Finally, progression toward invasive carcinoma is driven by *TP53* alterations [116].

The sessile serrated sequence begins in the proximal colon with a *BRAF* activating mutation that alters the MAPK-ERK pathway, inducing apoptosis arrest, colonocyte proliferation and *p16* and *IGFBP7* over-expression (Figure 3) [30]. These changes determine the transformation of the normal mucosa into hyperplastic crypts, which then, due to *p16* and *IGFBP7* promoter hypermethylation, evolve toward sessile serrated adenoma. In particular, HP lesions can stay dormant for a long time due to oncogene-induced senescence which inhibits the tumorigenic progress until an aberrant methylation of the *p16* and *IGFBP7* promoters subverts this protective mechanism. *P16* and *IGFBP7* are tumor suppressor genes: *IGFBP7* is a direct target and mediator of *p53*-dependent growth suppression, while *p16* encodes for the p16 protein that negatively regulates the *p16*/cyclin-dependent kinase/retinoblastoma gene pathway involved in the control of the cell cycle [117,118].

The oncogene-induced senescence process in CRC was first investigated in mice [119]. Then, the same mechanism has also been evidenced in human serrated polyps [118].

In human CRC, *p16* methylation is usually linked to CIMP-H and MSI phenotype, and is particularly associated with SSA/Ps with respect to HP and TSA tumors. In fact, in addition to the *BRAF*-mediated alteration of the MAPK pathway, the inactivating hypermethylation of *p16* and *IGFBP7* drives the progression of MVHP lesions toward sessile serrated adenomas/polyps, rendering the distinction between MVHP and SSA/P difficult to assess by endoscopy. Nevertheless, their distinction is clinically relevant due to their disparate outcome and relative interval of surveillance.

The next stage of polyp progression is the evolution of SSA/P lesion toward sessile serrated adenoma with high-grade dysplasia. This malignant evolution can be related either to *MLH1* aberrant methylation with consequent amplification of microsatellites (MSI), usually representative of a good prognosis, or to *MGMT* hypermethylation with a stable number of microsatellite (MSS), associated with the poorest prognosis (Figure 3) (Table 3) [29,31].

The serrated pathway has two end results that differ in their clinical and prognostic features as well as in their methylome profile: (i) the serrated adenocarcinoma (SAC) or (ii) the sporadic colorectal carcinoma showing histological and molecular features of MSI-H (hmMSI-H) [31,120]. The CRC subtype hmMSI-H is associated with a dense immune infiltrate and shows a better prognosis than SACs [120].

### A Distinct Variant of Colorectal Cancer: Serrated Adenocarcinoma

Serrated adenocarcinoma (SAC), which was first described by Jass and Smith in 1992, is histologically defined as an adenocarcinoma with serrated architecture and is one of the endpoints of the serrated pathway [121,122]. SAC accounts for 7.5–8.7% of all CRCs. It is located predominantly in the cecum and rectum, occurs particularly in females and has a worse prognosis than conventional carcinoma (CC) [121,123]. At the molecular level, SAC is a heterogeneous subtype. *KRAS* mutations, particularly the c12 G>A substitution, but predominantly *BRAF* alterations, are frequent [124]. Although SACs mainly arise from TSA lesions and show a MSS phenotype, about 20% of them originate from SSA/Ps and are MSI.

Several studies have indicated PTCH1, HIF1α and EPHB2 as SAC biomarkers [121,125,126,127]. PTCH1 expression is decreased, while HIF1α is increased in SACs in comparison to CC, evidencing the molecular and histopathological differences between the two classes of CRC [125]. *EPHB2,* which encodes a tyrosine kinase receptor, is one of the most studied genes related to SAC [128]. *EPHB2* is a well-established tumor suppressor gene whose downregulation often results from promoter hypermethylation [129]. The decreased expression of EPHB2 in serrated CRC may also potentiate the activation of the Notch signaling pathway which can further stimulate the expression of EPHB4 [128]. *EPHB2* frameshift mutations are also frequent in MSI adenomas and carcinomas. Furthermore, a novel biomarker of SAC has recently been reported and consists of fascin-1 (FSCN1) whose over-expression has been associated with a progression from adenoma to carcinoma [127,130].

Methylome profiling has been performed in order to identify some genes that are differentially methylated between SAC and CC. *DIO3* and *FOXD2* appeared more methylated and less expressed at the mRNA level in SAC than in CC, thus revealing a SAC-associated epigenetic signature [131]. Differences in the molecular signature between SAC and hmMSI-H have also been studied. *CD14* and *HLADOA* were the most significantly hypermethylated and underexpressed genes in hmMSI-H as compared to SAC [120]. In addition, gene expression profiling was carried out in order to clarify the role of the immune response between the two end results of the serrated pathway. As a result, a higher expression of *ICAM1*, an adhesion molecule involved in cellular immune responses, and a lower expression of *CRCP* and *CXCL14* were identified in SAC versus hmMSI-H CRCs [132].

Additional studies will be necessary to further characterize SAC at a molecular level, not only to better clarify the malignant process of CRC progression but also to identify novel biomarkers for SAC targeted therapy.

## 6. Superficially Serrated Adenoma

A novel subtype of CRC, namely superficially serrated adenoma, has been recently described [133]. It is characterized by mixed morphological features of both conventional and serrated adenomas.

Superficially serrated adenomas show a median size of 5 mm and are preferentially located in the sigmoid colon or rectum. They appear like SSA/P polyps. However, in some cases, they can look like larger and flat lesions. At the molecular level, superficially serrated adenomas are homogeneous. *KRAS* mutation, RSPO fusion/overexpression and *MLH1* loss are the most frequent alterations. *BRAF, APC* and *GNAS* mutations are present only in a few lesions. IHC analysis also revealed that superficially serrated adenomas show an overexpression of MYC, a nuclear accumulation of β-catenin and express Ki-67 preferentially in the upper half of the crypts.

Although identification of this novel type of polyp can contribute to better distinguish CRC subtypes, further studies will determine if superficially serrated adenoma can be subcategorized [133].

## 7. MicroRNAs and Long Non-Coding RNAs in Serrated Colorectal Pathway

MicroRNAs (miRNAs) and long non-coding RNAs (lncRNAs) are two important classes of RNAs involved in the regulation of gene expression [134]. Their altered expression has been described in CRCs, with distinct profiles observed between the conventional adenoma-carcinoma and serrated-carcinoma progression models [135,136,137]. Even if their role in cancer progression has not been elucidated yet, specific miRNA alterations have also been described in pre-cancerous lesions.

In this scenario, miR-31 is frequently overexpressed in sessile serrated adenomas suggesting an involvement in the progression of serrated lesions [138]. Interestingly, several studies showed an unique miRNA signature in different types of polyps. In this line, HPs and SSPs share a similar profile of miRNAs characterized by the down-regulation of a set of miRNAs, while normal colonic mucosa shows an up-regulation of miRNAs [139]. Another miRNA profiling in 109 patient biopsies allowed to discriminate between five different histopathologic groups [135]. In particular, the expression levels of miR-335, miR-222, and miR-214 were able to distinguish between non-serrated and serrated histology. Precisely, miR-335 was significantly overexpressed in non-serrated tissues compared to serrated lesions. Moreover, miR-222 and miR-214 were significantly downregulated in serrated polyps [135]. Finally, this study identified miR-125b and miR-320a as predictive biomarkers of the evolution toward CRC through the serrated pathway [135].

In a recent study, small RNA sequencing has been performed in 108 colon biopsies with different histology. Differential expression of miRNAs between tumor and healthy mucosal specimens was highlighted, and miRNAs specific of the serrated lesions were identified [136]. In particular, 23 miRNAs appeared differentially expressed between the serrated lesions and their paired healthy colon samples. Also, six miRNAs were differentially expressed between the serrated lesions and the hyperplastic polyps, with miR-31-5p being the most significantly modulated.

Similar results were observed for lncRNAs. Characterization of the lncRNA expression profiles in 888 CRC samples identified five different molecular clusters. These latter allowed to distinguish the conventional pathway from the serrated one [140]. Recently, the expression of 4898 lncRNAs has been monitored in 566 CRC samples and 282 lncRNAs illustrated the heterogeneity of CRCs [137]. Because the latter lncRNAs seem relevant for the development of CRC, their study may contribute to better understanding the origin of such tumor heterogeneity and facilitate the development of novel therapies [137].

Altogether, these findings show that non-coding RNAs may be useful to further discriminate the different subtypes of CRCs.

## 8. Dysbiosis of the Gut Microbiota: A New Biomarker?

About 39 trillion microorganisms colonize the adult gut system, resulting in a biomass of 0.2 kg [141]. Human gut microbiota, whose composition varies across gut segments and between individuals, plays a multitude of functions, such as structural and metabolic roles or homeostasis maintenance of the intestinal immune system which constitutes a natural barrier to pathogen infection [142,143].

The plethora of bacterial species found in the human gastrointestinal tract belong to three phyla: *Firmicutes* (30–50%) *Bacteroidetes* (20–40%) and *Actinobacteria* (1–10%) [144]. Environmental factors (infection, antibiotics, diet, lifestyle) can trigger an imbalance in the composition of the gut microbiota, known as dysbiosis. Dysbiosis has been associated with several diseases. In particular, it may initiate or promote carcinogenesis and influence anticancer immunosurveillance [144,145]. Thus, changes in microbiota composition are being evaluated as biomarkers for cancer diagnosis or prognosis [146,147]. 

The role of the gut microbiota in promoting or protecting against CRC has been studied. So far, these investigations have mainly been addressed to the conventional pathway, rather than the serrated one [148,149,150,151]. These analyzes particularly focused on the Gram-negative bacterium *Fusobacterium nucleatum (F. nucleatum)*, which is rarely present in the gut of healthy people [152].

The overabundance of *F. nucleatum* has been associated with CRC progression [153,154]. In this way, *F. nucleatum* is involved in mucosal inflammation and promotes colorectal carcinogenesis by modulating E-cadherin/beta-catenin signaling. Its detection has been related to particular CRC subtypes [155]. Precisely, *F. nucleatum* has been linked with CIMP and MSI CRCs, thus encouraging its exploitation as a prognostic biomarker [156].

Regarding the serrated pathway, it has been evidenced that *F. nucleatum* is present in 56% of CRCs but appears less frequent in premalignant lesions. *F. nucleatum* was not associated with serrated precursor polyps, but was related to CIMP-H status and to large tumors, comforting its role in CRC progression [157]. The study also demonstrated an increasing density of *F. nucleatum* in SSA/Ps along the path between the sigmoid and the cecum. This observation was explained by the anatomy of the crypts in SSA/P. These crypts appeared rich with mucinous cells producing high mucus content which favors the survival of the bacteria.

Another study by Park et al. investigated the composition of the gut microbiota, including the presence of *F. nucleatum,* in patients with tubular adenoma and SSA/P [158]. The abundance of *Fusobacteria* was comparable between the two subtypes of adenomas but lower in comparison to the CRC group. A similar observation was reported by Ito et al. leading to the conclusion that *Fusobacteria* could contribute to both conventional and serrated pathways [157,158].

The Human Microbioma Projects 1 and 2 (HMP1 and HMP2) directed by the National Institute of Health (NIH, Bethesda, MD, USA) aim at collecting and comparing the human microbioma in healthy and diseased conditions [159]. Available data already highlighted the great variability of the microbioma across different physiopathological and environmental conditions. Future investigations should benefit to precision medicine.

Thus, new studies will be necessary to correlate the gut microbiome profile with the serrated adenoma-carcinoma pathway, and to identify novel biomarkers predictive of specific serrated lesions.

## 9. Clinical Relevance of Molecular Alterations in the Serrated Colorectal Pathway

Molecular heterogeneity among the subtypes of serrated colorectal tumors translates into a variety of clinical and prognostic implications [19,160]. Precisely, clinicopathological features have been related to the presence of *BRAF* mutations, MSI and CIMP in serrated CRCs. Interestingly, geographical location seems contributing to such molecular heterogeneity. For instance, in contrast to Eastern populations, Western populations show prevalent *BRAF* mutated, CIMP, MSI CRCs [41].

The clinical role of MSI and CIMP has not been precisely described as opposed to *BRAF* and *KRAS* mutations. In general, *BRAF* mutated, MSI and CIMP CRCs are more frequent in females and often localized in the proximal colon [98,161]. MSI CRCs present a good prognosis, while *BRAF* mutated and CIMP tumors are related to a later age-of-onset and to a poor clinical outcome [38,65]. Serrated *BRAF* mutated tumors have also been associated with heavy smoking [162]. The latter shows a negative prognostic outcome especially in the late stages of the disease, is associated with aggressive phenotypes, and seems to favor metastases in the peritoneum rather than in the liver or lungs [163,164]. Metastatic *BRAF* mutated CRCs do not respond to anti-EGFR treatment, with or without chemotherapy [165]. Moreover, the presence of mutations in *BRAF* has not demonstrated a predictive value for CRC therapy, so far [161]. The 5-year disease-free survival rate and overall survival appear poor in *BRAF* mutated patients even after lung/liver metastasectomy [161,163].

*KRAS* mutations are clinically heterogeneous as illustrated by their involvement in both traditional and serrated CRC pathways. *KRAS* mutated tumors have been correlated with high body weight. An association between *KRAS* mutated tumors and female gender has also been raised in the literature but remains controversial [166,167]. *KRAS* mutated tumors have been mainly explored in metastatic CRCs and associated with a poor outcome, notably a low 5-year disease-free survival, but are also associated with a short cancer-specific survival in stage I CRCs [19,168].

CRCs harboring *KRAS* mutations also show resistance to anti-EGFR treatments and a negative response to 5-fluorouracil-based therapies [169]. In addition, *KRAS* mutations combined with MSS can increase the metastatic progression [170].

MSI is usually a molecular marker of a favorable prognosis independent of *BRAF*, *KRAS*, and CIMP status [19]. MSI is also associated with a lower frequency of late-stage diseases [171,172]. Patients with MSI cancers usually show a better prognosis and a longer disease-free survival with respect to MSS tumors [173]; they also benefit from chemotherapy [174]. However, patients with metastatic MSI CRCs having *BRAF* mutations show a poor survival rate [175]. The MSI test is recommended for patients with stage II CRC in order to evaluate chemotherapy-based strategies [176]. In fact, 5-fluorouracil treatment has no positive effect on survival in patients with MSI CRCs [177].

MSS cancer, developing from TSA or SSA/Ps, is related to a poor prognosis [172,178]. Several studies reported that MSS CRCs, associated with *BRAF* mutations, show higher mortality and decreased overall survival with respect to both MSI/*BRAF* mutated and MSS/*KRAS* mutated cancers [179,180,181]. The good prognosis of MSI/*BRAF* mutated cancers may be related to the increased immune response in MSI tumor [182].

CIMP tumors are clinically heterogeneous. CIMP-L CRCs are associated with male gender, while CIMP-Hs show a female preponderance and tend to manifest at a later age and are associated with cigarette smoking [73]. The prognostic value of CIMP remains controversial especially for metastatic CRCs, perhaps because of their high molecular heterogeneity. In fact, although CIMP is mostly reported as a negative prognostic factor in CRC patients, several studies demonstrated that its prognostic value can be influenced by *BRAF/KRAS* and MSI status [183,184,185]. Among them, CIMP-H, MSS, *BRAF* mutated tumors show the worst prognosis of all CRCs [19,22,186]. The role of CIMP in tumor response to therapy is also controversial [187]. Nevertheless, CIMP-H stage III CRC patients can benefit from adjuvant irinotecan plus 5-fluorouracil chemotherapy [188].

Finally, epigenetic alterations can also influence clinical outcome of CRC patients. Hypermethylation in *MLH1* or *MGMT* predicts good prognosis, whereas hypermethylation in *p16* or hypomethylation in the promoters of *LINE-1* and *IGF-2* are related to poor prognosis [19,113,189,190,191].

In conclusion, growing knowledge of the genetic mutations characterizing the different colorectal tumor subtypes, as well as their alterations at the epigenetic and signaling levels, is critical for better classifying CRCs, and therefore, for personalizing the treatments to reach superior efficacy (precision medicine).

## 10. Conclusions

This review is an excursus on the serrated pathway in colorectal carcinogenesis. It reports the new major genetic and epigenetic alterations, as well as the relevant emerging role of the gut microbiota, in serrated CRC. The molecular characterization of serrated polyps provides necessary information for their correct classification. Such classification allows to match the clinical signs and symptoms with the natural history of the disease; a critical consideration for adapting the care of the patient following diagnosis and during treatment follow-up.

Thus, we highlighted the need for combining a more precise set of molecular analyses together with histological assessments to better differentiate the serrated colorectal polyps.

In contrast to the conventional pathway, characterized by *KRAS* mutations, CIN, CIMP negative, and MSS, the progression from serrated polyp toward cancer seems driven and featured by alternative molecular imprints, with different prognostic significance.

Generally, there are two major serrated pathways, namely sessile and traditional, which differ in the lesions encountered along the oncogenic process: SSA/P and TSA, respectively. On the one hand, SSA/Ps are characterized by *BRAF* mutations, CIMP-H and *MLH1* promoter methylation. On the other hand, TSA lesions harbor *KRAS* or *BRAF* mutations and can be MSS, CIMP-H or CIMP-L.

To shed light on the malignant adenoma-to-carcinoma progression, several transcriptomic and epigenomic studies have demonstrated that serrated polyps display a different molecular expression profile compared to the CC, thus identifying, for each serrated lesion, panels of markers for subtype-specific molecular signature, including microRNAs and lncRNAs. Nevertheless, the classification of serrated CRCs, and their respective diagnostic and prognostic values, still remain to be clarified. As underlined above, such clarification is rendered difficult by the high heterogeneity and the undiscovered molecular features of serrated lesions. Thus, the definition of new specific genetic and epigenetic features will be useful for supporting clinical-decision making and driving the choice of the most appropriate therapeutic approach.

Further studies will be necessary to confirm the emerging role of the gut microbiota, to establish a commonly accepted molecular classification of serrated lesions and to identify novel targets for treating CRC. The availability of advanced technologies able to investigate the molecular bases of human diseases with high resolution will further improve this field. Among them, omic analyses at a single cell level (genomics, transcriptomics, proteomics, metabolomics, etc.), applicable to dissociated tumors or circulating tumor cells, will likely contribute to identify new biomarkers. All these advances will facilitate personalized endoscopic, histologic and molecular surveillance of serrated CRC lesions and ultimately improve their clinical management.

## Figures and Tables

**Table 1:** Morphologic categories and features of serrated colorectal lesions.

Histological Classification	Frequency (%) *	Location	Shape	Mucin Type	Size
Hyperplastic polyp (HP)	80–90%	Distal	Sessile, Flat	Variable	<5 mm
Microvesicular HP (MVHP)	60%	Distal	Sessile	Microvesicular	<5 mm
Goblet cell HP (GCHP)	30%	Distal	Sessile	Goblet cells	<5 mm
Mucin poor HP (MPHP)	10%	Distal	Sessile	Poor	<5 mm
Sessile serrated adenoma/polyp (SSA/P)	15–20%	Proximal	Sessile/Flat	Microvesicular	>5 mm
Traditional serrated polyp (TSA)	1–6%	Distal	Sessile/Pedunculated	Not present	>5 mm

* Frequency of all serrated polyps.

**Table 2:** Characteristics of CpG island methylator phenotype (CIMP) subtypes bearing serrated colorectal lesions.

CIMP Phenotype	CIMP-0	CIMP-L	CIMP-H
Location	Distal colon	Proximal colon	Proximal colon
Gender	no gender bias	Male	Female
Pathway	Conventional adenoma	Serrated or Conventional	Serrated adenoma
Gene mutations	*TP53* mutations	*KRAS*, *TP53* mutations	*BRAF* mutations
Epigenetic alterations	no *MLH1* methylation	no *MLH1* methylation	*MLH1* hypermethylation
MSI rate	MSS	MSS	MSI
CIN association	positive	positive	negative
Prognosis	Variable	High	Poor

**Table 3:** Molecular profile of serrated colorectal lesions.

Serrated Lesion	BRAF/KRAS Status	CIMP Rate	Gene Methylation	MSI Rate
HP	*BRAF* mutated	CIMP-H	*MLH1* not methylated	MSS
MPHP *	controversial	CIMP-H	controversial	controversial
GCHP *	*KRAS* mutated	CIMP-L	*MLH1* not methylated	MSS
MVHP *	*BRAF* mutated	CIMP-H	*MLH1* not methylated	MSS
SSA/P	*BRAF* mutated	CIMP-H	*MLH1* not methylated	MSS
SSAD	*BRAF* mutated	CIMP-H	*MLH1* hypermethylated	MSI
TSA	*KRAS/BRAF* mutated or neither	CIMP-L/-H	*MLH1* not methylated	MSS
TSA HGD	*KRAS* mutated	CIMP-L	*MGMT* hypermethylated	MSS

* MPHP, GCHP, MVHP are HP subtypes.
